# Sex differences in pharmacological treatment of major depressive disorder: results from the AMSP pharmacovigilance program from 2001 to 2017

**DOI:** 10.1007/s00702-021-02349-5

**Published:** 2021-05-11

**Authors:** Johanna Seifert, Fabienne Führmann, Matthias A. Reinhard, Rolf R. Engel, Xueqiong Bernegger, Stefan Bleich, Susanne Stübner, Eckart Rüther, Sermin Toto, Renate Grohmann, Marcel Sieberer, Waldemar Greil

**Affiliations:** 1 Department of Psychiatry, Social Psychiatry, and Psychotherapy, Hannover Medical School, Carl-Neuberg-Straße 1, 30625 Hannover, Germany; 2 Department of Psychiatry and Psychotherapy, KRH Psychiatrie GmbH, Wunstorf, Germany; 3 Department of Psychiatry and Psychotherapy, LMU University Hospital Munich, Munich, Germany; 4 Department of Forensic Psychiatry, Bezirksklinikum Ansbach, Ansbach, Germany; 5 Department of Psychiatry, Psychotherapy, and Psychosomatics, St. Marien-Hospital Hamm gGmbH, Hamm, Germany; 6 Prosomno, Clinic for Sleep Medicine, Munich, Germany; 7 Department of Psychiatry and Psychotherapy, University Witten/Herdecke, Witten, Germany; 8 Psychiatric Private Hospital, Sanatorium Kilchberg, Kilchberg, Switzerland

**Keywords:** Antidepressant drugs, Antipsychotic drugs, Drug safety, Psychopharmacotherapy, Gender, Depression

## Abstract

Data on drug prescription for outpatients with major depressive disorder (MDD) suggest women are more likely to be treated with psychotropic drugs, while data on sex differences regarding pharmacological treatment of psychiatric inpatients are currently not available. Drug utilization data from the program “Drug Safety in Psychiatry” (German: Arzneimittelsicherheit in der Psychiatrie, AMSP) of 44,418 psychiatric inpatients with MDD were analyzed for sex differences between 2001 and 2017. Sex differences were analyzed using relative risks (RR) and 95% confidence intervals (95% CI). Time trends were analyzed by comparing the first (2001–2003) with the last time period (2015–2017). In general, men and women were equally likely to use psychotropic drugs. Monotherapy was more common in men. Women were more likely to utilize ≥ 4 psychotropic drugs. Antidepressant drugs (ADDs) were the most prescribed drug class. Men had a higher utilization of noradrenergic and specific serotonergic antidepressants (RR 1.15; 95% CI 1.12–1.19), especially mirtazapine (RR 1.16; 95% CI 1.12–1.19), but also of other ADDs such as bupropion (RR 1.50; 95% CI 1.35–1.68). Males had a slightly higher utilization of second-generation antipsychotic drugs (RR 1.06; 95% CI 1.03–1.09) and were less often treated with low-potency first-generation antipsychotic drugs (RR 0.86; 95% CI 0.83–0.90). Tranquilizing (e.g., benzodiazepines; RR 0.89; 95% CI 0.86–0.92) and hypnotic drugs (e.g., Z-drugs; RR 0.85; 95% CI 0.81–0.89) were less utilized in the treatment of male patients. Not all sex differences were stable over time. More sex differences were detectable in 2015–2017 than in 2001–2003. Findings suggest that certain psychotropic drugs are preferred in the treatment of men vs. women, however, sex differences found in this study are not as large as in ambulatory settings. To make evidence-based sex-specific recommendations in the treatment of MDD, differences in drug response and tolerability need to be further researched.

## Introduction

Incidence of depression worldwide has increased by nearly 50% within the past three decades (Liu et al. 2019) with women remaining twice as likely to suffer from depression (Herzog et al. 2019). Women suffering from major depressive disorder (MDD) are more likely to have an earlier age of onset, develop subsequent depressive episodes, and present a chronic course of illness (Frackiewicz et al. 2000). Studies consistently report a higher utilization of antidepressant drugs (ADDs) among women within the ambulatory setting (Boyd et al. 2015; Sundell et al. 2011; Zhong et al. 2014; Serna et al. 2010; Van der Heyden et al. 2009; Estancial Fernandes et al. 2018). However, this pattern may not necessarily represent the psychiatric inpatient setting.

Biologically determined sex differences in brain and physiology play a relevant role in the development of MDD (Rubinow and Schmidt 2019), expression of clinical symptoms (Altemus et al. 2014) as well as in efficacy and tolerability of drugs used to treat MDD (Franconi and Campesi 2014; LeGates et al. 2019). The latter are currently not fully understood, in part due to a low rate of women’s participation in clinical trials in the past (Liu and Mager 2016). In the past, sex differences were largely ignored—the exclusion of women justified due to the complexity of the female hormonal cycle which would complicate investigations (Rubinow and Schmidt 2019). First implications of sex differences in the treatment of MDD arose in the late 1960s when T_3_ (l-triiodthyronine) was observed to be more effective in the augmentation of antidepressant treatment in women than men—a finding which encouraged further research (Khan et al. 2005). In the past decades, sex has been finding more extensive consideration in the treatment of MDD (Khan et al. 2005).

As of now, standardized guidelines on the sex-specific treatment of MDD are virtually unavailable. A recent review about sex differences in antidepressant response by LeGates et al. summarized that there is not a definite consensus on whether sex differences in antidepressant efficacy actually exist (LeGates et al. 2019). International guidelines on the treatment of MDD, such as the recommendations by the British Association for Psychopharmacology or the German S3 guideline by the German Association for Psychiatry, Psychotherapy, and Psychosomatics, merely suggest that women may benefit more from treatment with a selective serotonin reuptake inhibitor (SSRI) (DGPPN et al. 2017), while men may respond better to treatment with tricyclic antidepressants (TCAs) (Cleare et al. 2015; DGPPN et al. 2017). The National Institute for Health and Care Excellence (NICE) explicitly states, that little evidence supports prescribing patterns in relation to sex ((NICE) 2009).

The aim of this study was to assess the use of psychotropic drugs used in the treatment of patients suffering from MDD according to sex in a real-life clinical inpatient setting from 2001 to 2017. Because treatment of MDD is not limited to the use of ADDs, utilization of other psychotropic drug classes (i.e., antipsychotic drugs (APDs), antiepileptic drugs (AEDs), lithium (LI), tranquilizing drugs (TRDs), and hypnotic drugs (HYPDs)) and combination of drug classes will also find consideration. Furthermore, time trends in the sex-specific treatment of MDD are analyzed by comparing the first (2001–2003) with the last (2015–2017) time period to provide information on more recent utilization patterns and determine the temporal stability of sex differences.

## Methods

### Data source

This study used data pertaining to the utilization of psychotropic drugs by patients with MDD collected by the European program “Drug Safety in Psychiatry” (German: “Arzneimittelsicherheit in der Psychiatrie”, AMSP). Founded in 1993, AMSP has since gathered data on psychotropic drug use and severe adverse drug reactions (ADRs) from psychiatric hospitals within a real-life setting. The number of participating hospitals has increased from nine in 1994 to 52 psychiatric institutions in Germany, Austria, and Switzerland in 2017.

Drug use data are gathered on two reference days per year on which all participating hospitals document all drugs prescribed on these days including dosages along with further information on age, sex, as well as psychiatric and somatic illnesses of patients. Due to the inpatient setting, AMSP is able to assess actual utilization rates of psychotropic drugs versus merely prescription rates. A more detailed description of AMSP’s methods can be found elsewhere (Grohmann et al. 2004, 2014; Engel et al. 2004). Data evaluation and analysis of the AMSP database have been approved by both the Ethics Committee of the University of Munich and the Ethics Committee of the Hannover Medical School (Nr. 8100_BO_S_2018).

### Study population and design

All patients treated between 2001 and 2017 aged 18–100 years with a primary psychiatric diagnosis of MDD were included in the study. Other psychiatric comorbidities, such as anxiety disorders, PTSD, or substance abuse disorders, were not considered during analysis of data. Drug utilization data by sex on the reference days were included in further analyses. MDD was identified using the International Classification of Disease in its 10th Version (ICD–10) (WHO 1992) and categorized as mild (F32.00, F32.01, F33.0, F33.00, F33.01), moderate (F32.1, F32.10, F32.11, F33.1, F33.10, F33.11), and severe depression without (F32.2, F33.2) or with psychotic symptoms (F32.3, F33.3). By including all degrees of severity, this study aims to provide a comprehensive insight in the psychopharmacological treatment of all inpatients with MDD. Prior to 2018—and therefore including the entire data collection period—patients suffering from non-severe depression could receive inpatient care. This later changed after implementation of a new remuneration system which led to a tightening of admission criteria.

### Classification of psychotropic drugs

ADDs were categorized as follows:

- *SSRIs*: escitalopram, citalopram, sertraline*
- *Selective serotonin-norepinephrine reuptake inhibitors (SSNRIs)*: venlafaxine, duloxetine*
- *TCAs*: trimipramine, amitriptyline, doxepin*
- *Noradrenergic and specific serotonergic antidepressants (NaSSAs)*: mirtazapine*
- *Monoamine oxidase inhibitors (MAOIs)*: tranylcypromine, moclobemide**
- *Other ADDs*: trazodone, bupropion, agomelatine*

APDs were classified as “first-generation antipsychotics” (FGAs) or “second-generation antipsychotics” (SGAs). FGAs were sub-classified as “low potency” (lp) or “high potency” (hp).

- *lp FGAs*: pipamperone, promethazine, prothipendyl, melperone*
- *hp FGAs*: haloperidol, perazine, flupentixol**
- *SGAs*: quetiapine, olanzapine, risperidone, aripiprazole*

HYPDs primarily included the Z-drugs (zopiclone, zolpidem*). The group of TRDs mainly consisted of benzodiazepines (especially lorazepam, diazepam, oxazepam*). Finally, relevant AEDs included valproic acid, lamotrigine, and pregabalin*.

*Only drugs used in the treatment of ≥ 2.5%patients are listed.

**Because of very low overall utilization in these drug groups, drugs used in the treatment of ≥ 0.5% patients are listed.

### Statistical analysis

Sex-specific drug utilization was analyzed by calculating relative utilization rates between sexes as relative risks (RR) together with their 95% confidence intervals (95% CI). Changes in the sex-specific use of drugs over time were analyzed for two observation periods, namely 2001–2003 and 2015–2017. In addition to time-specific RRs, relative risk ratios (RRR) were calculated with their 95% confidence intervals to quantify the interaction term sex by time. RRs and RRRs were considered statistically significant when the null value (i.e., 1.0) was not included in the 95% CI.

## Results

### Sociodemographic and illness-related data according to sex

44,418 patients with a primary diagnosis of MDD were treated in the participating hospitals from 2001 to 2017. 62.7% of patients were female. Table 1 shows the distribution of characteristics (i.e., age groups, severity of MDD, number of psychotropic drugs) between sexes. Women were older than men (♀: 51.53 vs. ♂: 48.81 years). Severity of MDD showed only slight differences between sexes. Most common diagnosis among both men and women was severe depression without psychotic symptoms (♀: 58.6%; ♂: 58.2%), followed by moderate depression (♀: 27.9%; ♂: 27.5%), and severe depression with psychotic symptoms (♀: 12.4%; ♂: 12.9%). The diagnosis of mild depression was rare in this sample of inpatients. Most patients (♀: 96.2%; ♂: 95.3%) were treated with psychotropic drugs. Men were 23% more likely not to receive any psychotropic medication and 9% less likely to be treated with four or more psychotropic drugs than women (Table 1).

**Table 1:** Characteristics of the study population according to sex

	M		F		RR (95% CI)
	*N*	(%)	*N *	(%)	
Total	16,547	37.3	27,871	62.7	
Age in years (mean)	48.81		51.53		
Age groups					
< 31	2466	14.9	3585	12.9	1.15 (1.10–1.22)
31–60	10,396	62.8	16,019	57.5	1.09 (1.08–1.11)
61–90	3661	22.1	8197	29.4	0.75 (0.73–0.78)
> 91	24	0.1	70	0.3	0.58 (0.36–0.92)
Diagnosis					
Mild depression	236	1.4	314	1.1	1.27 (1.07–1.50)
Moderate depression	4543	27.5	7773	27.9	0.98 (0.95–1.02)
Severe depression	9636	58.2	16,326	58.6	0.99 (0.98–1.01)
Severe depression with psychosis	2132	12.9	3458	12.4	1.04 (0.99–1.09)
Pat. receiving any psychotropic drugs	15,764	95.3	26,799	96.2	0.99 (0.99–0.99)
N psychotropic drugs (mean)	2.60		2.68		
N psychotropic drugs					
0	783	4.7	1072	3.8	1.23 (1.12–1.35)
1	3553	21.5	5527	19.8	1.08 (1.04–1.12)
2	4923	29.8	8246	29.6	1.00 (0.98–1.03)
3	3847	23.2	6690	24.0	0.97 (0.94–1.00)
4 +	3441	20.8	6336	22.7	0.91 (0.88–0.95)

*N* number, *M* males, *F* females, *df* degrees of freedom, *pat*. patients, *RR* relative risk, *95% CI* 95% confidence interval

### Prescription trends of psychotropic drugs according to sex

#### Antidepressant drugs

ADDs were the most used psychotropic drug class; however, they were used less often in the treatment of both men and women in 2015–2017 (♂: 84.1%; ♀: 86.4%) than in 2001–2003 (♀: 89.1%; ♂: 90.0%). NaSSAs—mainly consisting of mirtazapine—were the ADD-subgroup with the greatest sex difference in utilization rates. NaSSAs were used more frequently in the treatment of men. In 2001–2003, use of mirtazapine was equal among both sexes, whereas utilization rates of mirtazapine were relevantly higher among male patients in 2015–2017 (Table 2).

**Table 2:** Use of antidepressant drugs from 2001 to 2017 and 2001 to 2003 and 2015 to 2017 among patients with MDD according to sex

	2001–2017					2001–2003					2015–2017					Sex differences over time
	M		F		M vs. F	M		F		M vs. F	M		F		M vs. F	2001–2003 vs. 2015–2017
	*N*	%	*N*	%	RR (95% CI)	*N*	%	*N*	%	RR (95% CI)	*N*	%	*N*	%	RR (95% CI)	RRR (95% C1)
*N* patients	16,547		27,871			1527		2863			3936		6304			
ADD																
Any ADD	14,431	87.2	24,794	89.0	0.98 (0.94–1.00)	1361	89.1	2578	90.0	0.99 (0.97–1.01)	3311	84.1	5445	86.4	0.97 (0.96–0.99)	0.98 (0.96–1.01)
SSRI	5154	31.1	9208	33.0	0.94 (0.92–0.97)	508	33.3	898	31.4	1.06 (0.97–1.16)	1199	30.5	2103	33.4	0.91 (0.86–0.97)	0.86 (0.77–0.96)
Escitalopram	1642	9.9	2913	10.5	0.95 (0.90–1.00)	54	3.5	81	2.8	1.25 (0.89–1.75)	369	9.4	753	11.9	0.78 (0.70–0.88)	0.63 (0.44–0.90)
Citalopram	1477	8.9	2684	9.6	0.93 (0.87–0.98)	210	13.8	400	14.0	0.98 (0.84–1.15)	194	4.9	317	5.0	0.98 (0.82–1.17)	1.00 (0.79–1.26)
Sertraline	1478	8.9	2356	8.5	1.06 (0.99–1.12)	124	8.1	229	8.0	1.02 (0.82–1.25)	570	14.5	823	13.1	1.11 (1.00–1.22)	1.09 (0.87–1.38)
SSNRI	4689	28.3	8323	29.9	0.95 (0.92–0.98)	277	18.1	494	17.3	0.98 (0.84–1.15)	1150	29.2	1947	30.9	0.95 (0.89–1.01)	0.90 (0.78–1.04)
Venlafaxine	3343	20.2	5737	20.6	0.98 (0.94–1.02)	275	18.0	486	17.0	1.06 (0.93–1.21)	741	18.8	1260	20.0	0.94 (0.87–1.02)	0.89 (0.76–1.04)
Duloxetine	1249	7.5	2424	8.7	0.87 (0.81–0.93)	0		0			348	8.8	607	9.6	0.92 (0.81–1.04)	
NaSSA	4882	29.5	7138	25.6	1.15 (1.12–1.19)	429	28.1	797	27.8	1.01 (0.91–1.11)	1046	26.6	1359	21.6	1.23 (1.15–1.32)	1.22 (1.08–1.38)
Mirtazapine	4809	29.1	7005	25.1	1.16 (1.12–1.19)	400	26.2	751	26.2	1.00 (0.90–1.10)	1042	26.5	1347	21.4	1.24 (1.15–1.33)	1.24 (1.09–1.41)
TCA	2081	12.6	4006	14.4	0.87 (0.83–0.91)	355	23.2	738	25.8	0.90 (0.81–1.01)	327	8.3	561	8.9	0.93 (0.82–1.06)	1.04 (0.87–1.23)
Trimipramine	647	3.9	1275	4.6	0.85 (0.80–0.94)	109	7.1	218	7.6	0.94 (0.75–1.17)	106	2.7	170	2.7	1.00 (0.79–1.27)	1.07 (0.77–1.48)
Amitriptyline	627	3.8	1100	3.9	0.96 (0.87–1.06)	78	5.1	141	4.9	1.04 (0.79–1.36)	125	3.2	187	3.0	1.07 (0.86–1.34)	1.03 (0.73–1.46)
Doxepin	390	2.4	776	2.8	0.84 (0.75–0.95)	82	5.4	173	6.0	0.89 (0.67–1.15)	55	1.4	109	1.7	0.81 (0.59–1.11)	0.91 (0.60–1.37)
MAOI	236	1.4	369	1.3	1.08 (0.92–1.27)	36	2.4	56	2.0	1.21 (0.80–1.82)	48	1.2	69	1.1	1.11 (0.77–1.61)	0.92 (0.53–1.61)
Other ADDs	2049	12.4	3590	12.9	0.96 (0.91–1.01)	112	7.3	204	7.1	1.03 (0.82–1.29)	661	16.8	1189	18.9	0.89 (0.82–0.97)	0.86 (0.68–1.10)
Trazodone	879	5.3	1720	6.2	0.86 (0.80–0.93)	33	2.2	51	1.8	1.21 (0.79–1.87)	295	7.5	656	10.4	0.72 (0.63–0.82)	0.59 (0.38–0.93)
Bupropion	578	3.5	647	2.3	1.50 (1.35–1.68)	0		0			232	5.9	249	3.9	1.49 (1.25–1.78)	
Agomelatine	389	2.4	811	2.9	0.81 (0.72–0.91)	0		0			105	2.7	232	3.7	0.72 (0.58–0.91)	

*M* males, *F* females, *N* number, *MDD* major depressive disorder, *ADD* antidepressant drug, *TCA* tricyclic antidepressant, *SSRI* selective serotonin reuptake inhibitor, *SSNRI* selective serotonin-norepinephrine reuptake inhibitor, *NaSSA* noradrenergic and specific serotonergic antidepressant, *MAOI* monoamine oxidase inhibitor, *RR* relative risk, *95% CI* 95% confidence interval, *RRR* relative risk ratio

Overall utilization of SSRIs was minimally higher among females, in particular in 2015–2017. This trend was especially noticeable for escitalopram, which was used more often in the treatment of MDD in 2015–2017 than 2001–2003 in general, and especially among women. Use of SSNRIs as a group did not show relevant sex differences at any time point. Duloxetine was used 13% less frequently in the treatment of men than women in 2001–2017 (Table 2).

Use of “other ADDs” greatly increased from 2001–2003 to 2015–2017. Within this heterogeneous group, trazodone was the most used drug. Rarely used in 2001–2003, trazodone was more commonly utilized in 2015–2017, especially among women. Data for bupropion and agomelatine were only available for the later timeframe. From 2015 to 2017, utilization of bupropion was almost 1.5 times higher among men, while men were less likely to be treated with agomelatine (Table 2).

TCAs were used slightly more often in the treatment of women from 2001 to 2017. Sex-specific utilization and time trends for MAOIs as well as for other individual ADDs can be found in Table 2.

#### Antipsychotic drugs

Almost half of men and women were treated with APDs from 2001 to 2017 (♂: 49.3%; ♀: 49.4%). APD-use was slightly higher among men in 2015–2017. Men were more likely to be treated with SGAs—this trend was especially apparent in 2001–2003 at which time SGAs were used in the treatment of 30.1% of men and 24.7% of women. In 2015–2017, SGA utilization rates were higher among both sexes (♂: 41.1%; ♀: 37.4%) than in 2001–2003 but did not differ as much between sexes. Similar patterns were found for the use of olanzapine. Utilization of quetiapine was low in 2001–2003, whereas its utilization was 6.5 times higher among women and eightfold higher among men in 2015–2017. At this time, men were more likely to be treated with quetiapine (Table 3).

**Table 3:** Use of antipsychotic drugs from 2001 to 2017 and 2001 to 2003 and 2015 to 2017 among patients with MDD according to sex

	2001–2017					2001–2003					2015–2017					Sex differences over time
	M		F		M vs. F	M		F		M vs. F	M		F		M vs. F	2001–2003 vs. 2015–2017
	*N*	%	*N*	%	RR (95% CI)	*N*	%	*N*	%	RR (95% CI)	*N*	%	*N*	%	RR (95% CI)	RRR (95% C1)
*N* patients	16,547		27,871			1527		2863			3936		6304			
APD																
Any APD	8162	49.3	13,761	49.4	1.00 (0.98–1.02)	694	45.4	1250	43.7	1.04 (0.97–1.12)	2028	51.5	3062	48.6	1.06 (1.02–1.10)	1.02 (0.94–1.10)
SGA	6117	37.0	9742	35.0	1.06 (1.03–1.06)	460	30.1	708	24.7	1.22 (1.10–1–35)	1619	41.1	2360	37.4	1.10 (1.05–1.15)	0.90 (0.81–1.01)
Quetiapine	2973	18.0	4815	17.3	1.04 (1.00–1.08)	42	2.8	92	3.2	0.86 (0.60–1.23)	906	23.0	1313	20.8	1.11 (1.03–1.19)	1.29 (0.89–1.86)
Olanzapine	1544	9.3	2238	8.0	1.16 (1.09–1.24)	219	14.3	302	10.5	1.36 (1.16–1.60)	313	8.0	416	6.6	1.21 (1.05–1.39)	0.89 (0.71–1.10)
Risperidone	1107	6.7	1727	6.2	1.09 (1.01–1.17)	130	8.5	206	7.2	1.18 (0.96–1.46)	284	7.2	424	6.7	1.07 (0.93–1.24)	0.91 (0.70–1.17)
Aripiprazol	397	2.4	746	2.7	0.90 (0.79–1.01)	0		0			166	4.2	297	4.7	0.90 (0.74–1.08)	
FGA lp	2714	16.4	5271	18.9	0.86 (0.83–0.90)	233	15.3	535	18.7	0.82 (0.71–0.94)	594	15.1	1079	17.1	0.88 (0.80–0.97)	1.08 (0.91–1.28)
Pipamperone	779	4.7	1474	5.3	0.89 (0.82–0.97)	45	2.9	113	3.9	0.75 (0.53–1.05)	223	5.7	386	6.1	0.93 (0.79–1.09)	1.24 (0.85–1.80)
Promethazine	552	3.3	1158	4.2	0.80 (0.73–0.87)	48	3.1	125	4.4	0.72 (0.52–1.00)	104	2.6	239	3.8	0.70 (0.56–0.87)	0.97 (0.65–1.44)
Prothipendyl	599	3.6	1109	4.0	0.91 (0.83–1.00)	42	2.8	85	3.0	0.93 (0.64–1.33)	157	4.0	268	4.3	0.95 (0.77–1.14)	1.01 (0.67–1.53)
Melperone	357	2.2	848	3.0	0.71 (0.63–0.80)	38	2.5	111	3.9	0.64 (0.25–0.92)	55	1.4	104	1.6	0.85 (0.61–1.17)	1.32 (0.81–2.15)
FGA hp	499	3.0	911	3.3	0.92 (0.83–1.03)	114	7.5	189	6.6	1.13 (0.90–1.41)	62	1.6	108	1.7	0.92 (0.67–1.25)	0.81 (0.55–1.19)

*M* males, *F* females, *N* number, *MDD* major depressive disorder, *APD* antipsychotic drug, *FGA* first-generation antipsychotic drug, *SGA* second-generation antipsychotic drug, *lp* low potency, *hp* high potency, *RR* relative risk, *95% CI* 95% confidence interval, *RRR* relative risk ratio

Utilization of lp FGAs in general as well as of the four most commonly used individual substances (i.e., pipamperone, promethazine, prothipendyl, melperone) was lower in men overall as well as in both time periods. Use of hp FGAs did not show any relevant sex differences (Table 3).

#### Tranquilizing and hypnotic drugs

TRDs were the third-most utilized drug class. Men were 11% less likely to be treated with TRDs (♂: 27.2%; ♀: 30.5%). TRD use decreased from 2001–2003 to 2015–2017. While in 2001–2003, utilization of TRD did not show a sex difference, women had slightly higher utilization rates in 2015–2017. Lorazepam was the most prescribed TRD, also with lower use among male patients. Use of diazepam, much less used than lorazepam overall, further decreased in 2015–2017 and was used more by men (Table 4).

**Table 4:** Use of tranquilizing, hypnotic, and epileptic drugs and lithium from 2001 to 2017 and 2001 to 2003 and 2015 to 2017 among patients with MDD according to sex

	2001–2017					2001–2003					2015–2017					Sex differences over time
	M		F		M vs. F	M		F		M vs. F	M		F		M vs. F	2001–2003 vs. 2015–2017
	*N*	%	*N*	%	RR (95% CI)	*N*	%	*N*	%	RR (95% CI)	*N*	%	*N*	%	RR (95% CI)	RRR (95% C1)
*N* patients	16,547		27,871			1527		2863			3936		6304			
TRD																
Any TRD	4498	27.2	8497	30.5	0.89 (0.86–0.92)	505	33.1	1019	35.6	0.93 (0.85–1.01)	910	23.1	1623	25.7	0.90 (0.84–0.96)	0.97 (0.86–1.08)
Lorazepam	3056	18.5	5575	20.0	0.92 (0.89–0.96)	310	20.3	625	21.8	0.93 (0.82–1.05)	629	16.0	1073	17.0	0.94 (0.86–1.03)	1.01 (0.87–1.17)
Diazepam	534	3.2	944	3.4	0.95 (0.86–1.06)	64	4.2	158	5.5	0.76 (0.57–1.01)	72	1.8	92	1.5	1.25 (0.92–1.70)	1.65 (1.06–2.50)
Oxazepam	419	2.5	868	3.1	0.81 (0.72–0.91)	62	4.1	109	3.8	1.07 (0.79–1.45)	93	2.4	187	3.0	0.80 (0.62–1.02)	0.75 (0.50–1.11)
HYPD																
Any HYPD	2266	13.7	4491	16.1	0.85 (0.81–0.89)	338	22.1	672	23.5	0.94 (0.84–1.06)	351	8.9	755	12.0	0.74 (0.66–0.84)	0.79 (0.67–0.93)
Zopiclone	1080	6.5	2017	7.2	0.90 (0.84–0.97)	95	6.2	204	7.1	0.87 (0.69–1.10)	135	3.4	270	4.3	0.80 (0.65–0.98)	0.92 (0.67–1.25)
Zolpidem	700	4.2	1415	5.1	0.83 (0.76–0.91)	161	10.5	315	11.0	0.96 (0.80–1.17)	119	3.0	233	3.7	0.82 (0.66–1.02)	0.85 (0.64–1.13)
AED																
Any AED	2348	14.2	4255	15.3	0.92 (0.88–0.97)	205	13.4	337	11.8	1.14 (0.97–1.34)	506	12.9	1011	16.0	0.80 (0.73–0.89)	0.70 (0.58–0.85)
Pregabalin	751	4.5	1483	5.3	0.85 (0.78–0.93)	0		0			240	6.1	532	8.4	0.72 (0.62–0.83)	
Lamotrgine	393	2.4	945	3.4	0.70 (0.62–0.79)	20	1.3	37	1.3	1.0.1 (0.59–1.74)	81	2.1	220	3.5	0.59 (0.45–0.76)	0.58 (0.32–1.06)
Valproic acid	539	3.3	692	2.5	1.31 (1.17–1.47)	51	3.3	90	3.1	1.06 (0.76–1.49)	72	1.8	78	1.2	1.48 (1.08–2.03)	1.39 (0.88–2.21)
Lithium																
Any LI salt	969	5.9	1436	5.2	1.14 (1.05–1.23)	136	8.9	214	7.5	1.19 (0.97–1.46)	205	5.2	293	4.6	1.12 (0.94–1.33)	0.94 (0.72–1.23)

*M* males, *F* females, *N* number, *MDD* major depressive disorder, *TRD* tranquilizing drug, *HYPD* hypnotic drug, *AED* antiepileptic drug, *LI* lithium, *RR* relative risk, *95% CI* 95% confidence interval, RRR relative risk ratio

HYPDs were the fourth-most used drug class from 2001 to 2017 and were used less frequently in the treatment of men. Sex-related use of HYPDs did not differ in 2001–2003. In 2015–2017, men had a 26% lower HYPD utilization than women and therefore comprised the drug class with the greatest sex difference in 2015–2017 (Table 4).

#### Antiepileptic drugs and lithium

Overall AED utilization was slightly higher among females, especially in the later time period. In 2015–2017, men were less likely to be treated with lamotrigine and pregabalin. The sex ratio was reversed for the use of lithium. Lithium salts were the least utilized psychotropic drug class. Men had 14% higher utilization rates of lithium from 2001 to 2017 (Table 4).

### Trends in polypsychopharmacy

Concurrent utilization of ADD + APD was the most common drug combination among men and women and used by half of all male and female ADD users (♂: 50.3%; ♀: 50.1%). ADD + APD was slightly more often used in treatment of men in 2015–2017. Second-most common combination was the concomitant utilization of two ADDs (♂: 32.0%; ♀: 31.1%) which did not show any relevant sex-specific trends. Use of ADD + TRD (♂: 28.0%; ♀: 31.0%) was the third-most common drug combination. Utilization of ADD + TRD was higher among females from 2001 to 2017 and 2015 to 2017. The combination of HYPDs and AEDs with ADDs both showed a higher utilization among women in 2015–2017 without any clear sex differences in 2001–2003 (Table 5**)**.

**Table 5:** Use of combinations of psychotropic drug classes from 2001 to 2017 and 2001 to 2003 and 2015 to 2017 among patients with MDD according to sex

	2001–2017					2001–2003					2015–2017					Sex differences over time
	M		F		M vs. F	M		F		M vs. F	M		F		M vs. F	2001–2003 vs. 2015–2017
	*N*	%	*N*	%	RR (95% CI)	*N*	%	*N*	%	RR (95% CI)	*N*	%	*N*	%	RR (95% CI)	RRR (95% C1)
*N* patients	16,547		27,871			1527		2863			3936		6304			
Combination with any ADD (% of pat. treated with ADD)																
N pat. with ADD	14,431		24,794			1361		2578			3311		5445			
ADD +																
ADD	4614	32.0	7717	31.1	1.03 (1.00–1.06)	349	25.6	616	23.9	1.07 (0.96–1.20)	1121	33.9	1765	32.4	1.04 (0.98–1.11)	0.97 (0.86–1.11)
APD	7257	50.3	12433	50.1	1.00 (0.98–1.02)	637	46.8	1138	44.1	1.06 (0.99–1.14)	1753	52.9	2719	49.9	1.06 (1.02–1.11)	1.00 (0.92–1.09)
TRD	4041	28.0	7683	31.0	0.90 (0.88–0.93)	470	34.5	934	36.2	0.95 (0.87–1.04)	792	23.9	1441	26.5	0.90 (0.84–0.97)	0.95 (0.84–1.07)
HYPD	2056	14.2	4107	16.6	0.86 (0.82–0.90)	309	22.7	612	23.7	0.96 (0.85–1.08)	312	9.4	663	12.2	0.77 (0.68–0.88)	0.81 (0.68–0.96)
LI	904	6.3	1325	5.3	1.17 (1.08–1.27)	126	9.3	196	7.6	1.22 (0.98–1.51)	195	5.9	276	5.1	1.16 (0.97–1.39)	0.95 (0.72–1.26)
AED	2042	14.2	3825	15.4	0.91 (0.87–0.96)	185	13.6	306	11.9	1.15 (0.97–1.36)	433	13.1	909	16.7	0.78 (0.70–0.87)	0.68 (0.56–0.84)
Combination of ADD with (% of pat. treated with ADD)																
ADD +																
ADD + APD	2356	16.3	3748	15.1	1.08 (1.03–1.13)	158	11.6	265	10.3	1.13 (0.94–1.36)	599	18.1	862	15.8	1.14 (1.04–1.26)	1.01 (0.82–1.25)
APD + APD	1305	9.0	2422	9.8	0.93 (0.86–0.99)	118	8.7	187	7.3	1.20 (0.96–1.49)	296	8.9	593	10.9	0.82 (0.72–0.94)	0.69 (0.53–0.89)
APD + TRD	2373	16.4	4465	18.0	0.91 (0.87–0.96)	255	18.7	454	17.6	1.06 (0.93–1.22)	486	14.7	860	15.8	0.93 (0.84–1.03)	0.87 (0.74–1.04)
APD + HYPD	1119	7.8	2111	8.5	0.91 (0.85–0.98)	143	10.5	253	9.8	1.07 (0.88–1.30)	191	5.8	367	6.7	0.86 (0.72–1.01)	0.80 (0.62–1.03)

*M* males, *F* females, *N* number, *MDD* major depressive disorder, *ADD* antidepressant drug, *APD* antipsychotic drug, *TRD* tranquilizing drug, *HYPD* hypnotic drug, *AED* antiepileptic drug, *LI* lithium, *RR* relative risk, *95% CI* 95% confidence interval, *RRR* relative risk ratio

#### ADD and concomitant utilization of lp FGAs

Concomitant use of lp FGAs with different ADD-subgroups was lower among men during the overall observation period, especially for lp FGA + SSRI and lp FGA + NaSSA. However, sex differences for lp FGA + SSRI and lp FGA + NaSSA were no longer detectable in 2015–2017; thus, this drug combination had the most significant change in sex differences from 2001–2003 to 2015–2017. In 2015–2017, use of lp FGA + SSNRI was the only combination of ADD-subgroup with lp FGAs with a sex difference showing higher utilization among women (Table 6).

**Table 6:** Use of antidepressant drug classes in combinations with other psychotropic drugs from 2001 to 2017 and 2001 to 2003 and 2015 to 2017 among patients with MDD according to sex

	2001–2017					2001–2003					2015–2017					Sex differences over time
	M		F		M vs. F	M		F		M vs. F	M		F		M vs. F	2001–2003 vs. 2015–2017
	*N*	%	*N*	%	RR (95% CI)	*N*	%	*N*	%	RR (95% CI)	*N*	%	*N*	%	RR (95% CI)	RRR (95% C1)
*N* patients	16547		27871			1527		2863			3936		6304			
Combination with SSRI (% of pat. treated with SSRI)																
N pat. with SSRI	5154		8323			508		898			1199		2103			
SSRI +																
NaSSA	1059	20.5	1599	19.2	1.07 (1.00–1.15)	70	13.8	113	12.6	1.10 (0.83–1.45)	238	19.8	330	15.7	1.27 (1.09–1.47)	1.16 (0.84–1.58)
SGA	1838	35.7	2937	35.3	1.01 (0.96–1.06)	167	32.9	207	23.1	1.43 (1.20–1.69)	498	41.5	727	34.6	1.20 (1.10–1.31)	0.84 (0.69–1.02)
lp FGA	846	16.4	1682	20.2	0.81 (0.75–0.88)	77	15.2	158	17.6	0.86 (0.67–1.11)	180	15.0	315	15.0	1.00 (0.85–1.19)	1.16 (0.86–1.57)
TRD	1334	25.9	2640	31.7	0.82 (0.77–0.86)	148	29.1	288	32.1	0.91 (0.77–1.07)	275	22.9	534	25.4	0.90 (0.80–1.03)	0.99 (0.81–1.23)
HYPD	680	13.2	1403	16.9	0.78 (0.72–0.85)	105	20.7	193	21.5	0.96 (0.78–1.19)	91	7.6	232	11.0	0.69 (0.55–0.87)	0.72 (0.52–0.98)
Combination with SSNRI (% of pat. treated with SSNRI)																
N pat. with SSNRI	4689		9208			277		494			1150		1947			
SSNRI +																
NaSSA	1268	27.0	1825	19.8	1.36 (1.28–1.45)	69	24.9	97	19.6	1.27 (0.97–1.66)	325	28.3	406	20.9	1.36 (1.19–1.53)	1.07 (0.79–1.44)
SGA	2099	44.8	3521	38.2	1.17 (1.12–1.22)	106	38.3	147	29.8	1.29 (1.05–1.57)	548	47.7	874	44.9	1.06 (0.98–1.15)	0.83 (0.66–1.02)
lp FGA	830	17.7	1780	19.3	0.92 (0.85–0.99)	43	15.5	104	21.1	0.73 (0.53–1.02)	191	16.6	400	20.5	0.81 (0.69–0.95)	1.10 (0.77–1.57)
TRD	1367	29.2	2653	28.8	1.01 (0.96–1.07)	124	44.8	211	42.7	1.05 (0.89–1.24)	289	25.1	533	27.4	0.92 (0.81–1.04)	0.88 (0.71–1.08)
HYPD	666	14.2	1375	14.9	0.95 (0.87–1.04)	61	22.0	110	22.3	0.99 (0.75–1.30)	111	9.7	251	12.9	0.75 (0.61–0.92)	0.76 (0.53–1.07)
Combination with NaSSA (% of pat. treated with NaSSA)																
N pat. with NaSSA	4882		7138			429		797			1046		1359			
NaSSA +																
SGA	1708	35.0	2388	33.5	1.05 (0.99–1.10)	118	27.5	188	23.6	1.17 (0.96–1.42)	416	39.8	501	36.9	1.08 (0.97–1.19)	0.93 (0.74–1.16)
lp FGA	771	15.8	1287	18.0	0.88 (0.81–0.95)	60	14.0	170	21.3	0.66 (0.50–0.86)	165	15.8	204	15.0	1.05 (0.87–1.27)	1.60 (1.15–2.23)
TRD	1547	31.7	2483	34.8	0.91 (0.86–0.96)	162	37.8	330	41.4	0.91 (0.79–1.06)	295	28.2	407	29.9	0.94 (0.83–1.07)	1.03 (0.85–1.25)
HYPD	670	13.7	1388	19.4	0.71 (0.65–0.77)	101	23.5	207	26.0	0.91 (0.74–1.11)	103	9.8	175	12.9	0.76 (0.61–0.96)	0.84 (0.62–1.15)

*M* males, *F* females, *N* number, *MDD* major depressive disorder, *APD* antipsychotic drug, *SSRI* selective serotonin reuptake inhibitor, *SSNRI* selective serotonin-norepinephrine reuptake inhibitor, *NaSSA* noradrenergic and specific serotonergic antidepressant, *MAOI* monoamine oxidase inhibitor, *FGA* first-generation antipsychotic drug, *SGA* second-generation antipsychotic drug, *lp* low potency, *RR* relative risk, *95% CI* 95% confidence interval, *RRR* relative risk ratio

#### ADD and concomitant utilization of SGAs

Utilization of SGA + ADD increased for all ADD subgroups (i.e., SSRIs, SSNRIs, NaSSAs) from 2001–2003 to 2015–2017. Concomitant treatment with an SGA was highest among SSNRI users during both the entire observation period, 2001–2003, and 2015–2017. This combination was used more in male patients overall and in 2001–2003 but no longer in 2015–2017. Use of SSRI + SGA was used more in the treatment of men in 2001–2003 and 2015–2017 whereas no sex differences were observed for NaSSA + SGA (Table 6).

#### Concomitant utilization of two antidepressant drugs

Male SSNRI users were 36% more likely to also be treated with NaSSAs than females from 2001 to 2017. This was also the case in 2015–2017. The combined use of SSRI + NaSSA did not show a sex difference from 2001 to 2017. However, this combination of ADDs was used more in the treatment of male SSRI users than females in 2015–2017 (Table 6).

#### ADD and concomitant utilization of TRDs and HYPDs

When examining the overall time period, concomitant use of TRDs was 9% lower among male NaSSA users and 18% lower among male SSRI users. Sex differences were not detectable in 2001–2003 or 2015–2017. Utilization of SSNRI + TRD did not show sex differences overall or at any time point (Table 6).

From 2001 to 2017, HYPD use was 29% lower among lower among male NaSSA users and 22% lower among male SSRI users. Concomitant use of HYPDs with SSRIs, SSNRIs, and NaSSAs was higher among female patients in 2015–2017. In 2001–2003, use of these drug combinations did not differ between sexes. SSRI + HYPD was the combination with the most prominent sex difference (Table 6).

#### Most used combinations of two psychotropic drugs

The most frequently used combination and with a 7% higher utilization among men was mirtazapine + lorazepam (2802 patients from 2001 to 2017, i.e., 22.7% of male mirtazapine users vs. 24.4% of female mirtazapine users; RR 0.93; 95% CI 0.87–0.99). Second-most common was the concomitant use of venlafaxine + mirtazapine (2270 patients; i.e., 28.1% of male venlafaxine users vs. 23.2% of female venlafaxine users). Male venlafaxine users were 21% more likely to receive this drug combination (95% CI 1.13–1.30). A sex difference was not detectable when considering this combination from the perspective of mirtazapine users—venlafaxine was concomitantly used by 19.5% of male and 19.0% of female mirtazapine users. Third-most common drug combination was venlafaxine + quetiapine (2002 patients) used by 22.0% and 22.1% of male and female venlafaxine users, respectively.

#### Most used combination of three psychotropic drugs

Nearly one-fourth of women and men were treated with three psychotropic drugs (Table 1). The most common triple psychotropic drug class combination among both men and women was ADD + APD + TRD. Men had a lower risk of being treated with this combination in 2001–2017, however, utilization of this drug group combination was equal among men and women in 2015–2017. The second-most common triple combination among both sexes was the use of two ADDs and an APD—a drug combination more frequently used in the treatment of male ADD users than females. This combination was more utilized in 2015–2017, at which time utilization was also higher among men (Table 5).

Venlafaxine, mirtazapine, and lorazepam were the most used combination of individual drugs (605 patients; i.e., 7.3% of male venlafaxine users vs. 6.3% of female users or 5.1% of male mirtazapine users vs. 5.2% of female users). Mirtazapine, quetiapine, and lorazepam were used by 570 patients (4.9% of male mirtazapine users vs. 4.8% of female users), followed by venlafaxine, mirtazapine, and quetiapine (500 patients; i.e., 4.6% of male mirtazapine users vs. 4.0% of female users or 6.6% of male venlafaxine users vs. 4.9% of female users). None of the most common triple combinations showed relevant sex differences.

## Discussion

The present study focuses on sex differences of psychotropic drug utilization in the treatment of psychiatric inpatients with MDD over a 17-year period. In order to detect time trends in sex-specific drug utilization, the timeframes 2001–2003 and 2015–2017 were also analyzed. To the best of our knowledge, this is the first study investigating drug utilization trends of patients with MDD with special attention to sex differences over time within the inpatient psychiatric setting. A more detailed analysis of time trends in the utilization of psychotropic drugs during this time period can be found elsewhere (Seifert et al. 2021). In brief, overall utilization of ADDs decreased slightly during this timeframe. TCAs were used less frequently in 2015–2017, whereas utilization of SSRIs and “other ADDs” increased. More patients were treated with a combination of two ADDs in 2015–2017 than 2001–2003. Further, APDs, especially SGAs, appeared to “replace” the use of TRDs and HYPDs (Seifert et al. 2021).

### Sex differences in psychotropic drug utilization

Utilization of psychotropic drugs is associated with a patient’s likeliness to seek medical care (Subramaniam et al. 2013), a factor that has been eliminated in inpatient setting in which treatment has already been sought. The present study was able to confirm some of the observations other researchers have made, however, relative risks for the use of psychotropic drug groups were usually not as discrepant among this collective of inpatients as among the outpatient collectives analyzed by other authors. For example, while Serna et al. found that Spanish men were about 60% less likely to use any psychotropic drug than women (Serna et al. 2010), over 95% of both male and female inpatients examined in this study were treated with at least one psychotropic drug. A majority of currently available studies have found higher psychotropic drug utilization among women within the ambulatory setting (Sundell et al. 2011; Luo et al. 2020; Zhong et al. 2014; Estancial Fernandes et al. 2018; Serna et al. 2010; Boyd et al. 2015)—a plausible finding when considering that common mental disorders such as MDD are more prevalent in women (Herzog et al. 2019). The high utilization of psychotropic drugs among inpatients allows a more differentiated look at sex-related utilization trends of psychotropic drug classes and individual drugs, which previous studies have rarely commented on.

Population-based RRs for the utilization of ADDs (Boyd et al. 2015; Van der Heyden et al. 2009; Estancial Fernandes et al. 2018; Sundell et al. 2011; Zhong et al. 2014; Serna et al. 2010; Luo et al. 2020; Yu et al. 2020) by women has been noted to be up to 2.42 (Serna et al. 2010) times higher than by men. Studies considering subclasses of ADDs found that women were more likely to be treated with SSRIs (Sundell et al. 2011; González-López et al. 2015) and TCAs (Sundell et al. 2011). The study which presents the most detailed description of sex-specific drug utilization patterns among outpatients with MDD was performed by Sundell et al. They found that citalopram and sertraline followed by venlafaxine and mirtazapine were the most frequently prescribed ADDs among a Swedish outpatient population of young adults aged 20–34 years. Men were more than twice as likely to be prescribed mirtazapine (RR 2.22), while women had a 10% higher chance of being prescribed SSRIs, especially fluoxetine (RR 1.70), and were 14% more likely to use TCAs. Among SSNRIs, use of duloxetine was 13% lower among men, whereas men had 21% higher odds of being prescribed venlafaxine (Sundell et al. 2011).

In the present study, the greatest sex difference in ADD-utilization was found for NaSSAs. NaSSAs were also the only ADD subgroup which were used more often in the treatment of men than women (RR 1.16 from 2001 to 2017). Female patients were treated with TCAs more often during the overall observation period, however, this trend was not detectable at either time point. Utilization of SSRIs was higher among women during the later time period, whereas a sex difference was not detectable in 2001–2003. The shift to higher utilization of SSRIs in the treatment of women is potentially due to the application of the German S3 guidelines in which the higher efficacy of SSRIs in the treatment of women is mentioned without being definitively recommended (DGPPN et al. 2017).

Sex differences in the use of anxiolytic drugs are reported inconsistently. Several authors were unable to detect significant sex differences in the dispensation of benzodiazepines (Van der Heyden et al. 2009; Estancial Fernandes et al. 2018; Subramaniam et al. 2013). Among the general population, Boyd et al. found German women were more likely to be treated with benzodiazepines. When adjusted for prevalence of anxiety disorders and MDD, utilization of benzodiazepines did not show any sex differences in the German ambulatory setting (Boyd et al. 2015). Higher benzodiazepine use was found among Portuguese, Bulgarian, French, and Spanish women with MDD (Boyd et al. 2015). The results presented here show that utilization of HYPDs and TRDs has shown significant changes during this study’s 17-year observation period. Use of both drug groups has decreased from 2001–2003 to 2015–2017. Synchronously, sex differences have appeared. In more recent years, women have been more likely to be treated with these drug groups, especially HYPDs.

Sex differences for the prescription of APDs and mood-stabilizing drugs, such as AEDs and lithium, among patients with MDD are largely unavailable. A study examining the population-based prevalence of psychotropic drug use over a 12-month period in ten European countries found that the use of APDs and mood-stabilizing drugs did not show any significant sex differences among patients with mood disorders in any country (Boyd et al. 2015). Results presented in this study show that alongside a decline in TRD and HYPD utilization from 2001–2003 to 2015–2017, overall use of APDs increased. This may signify that TRDs and HYPDs are being replaced by other psychotropic drug classes that do not carry a risk for dependency. This seems to be the case especially among male patients. While TRD and HYPD use was not sex-dependent in 2001–2003, this study revealed in particular a relevantly higher (i.e., 25%) HYPD utilization among women in 2015–2017. Compared to this, APD utilization among men only showed a disproportionately small increase (i.e., 6%) indicating that there may also be further underlying considerations, such as ADRs, that decidedly affect selection of drug prescriptions.

Sundell et al. found that men were significantly more likely to be treated with AEDs, such as carbamazepine, lamotrigine, and valproic acid, whereas the use of lithium showed no sex-related trends (Sundell et al. 2011). Higher use of lithium has been described among male patients with bipolar disorder, while women were more often treated with lamotrigine (Karanti et al. 2015). The same study found similar prescription rates of APDs and other mood-stabilizing agents including valproic acid among sexes (Karanti et al. 2015). In this study, valproic acid showed a very prominent utilization trend of preferred use among male patients—a finding that is not surprising under consideration of the drug’s teratogenic properties as well as the risk for hormonal abnormalities (Gotlib et al. 2017). This sex-specific recommendation found more consideration in 2015–2017 at which point utilization of valproic acid was lower and utilization of lamotrigine was higher in the treatment of female patients.

### Sex differences in response to psychotropic drugs

Undeterred by years of research, recommendations for sex-specific treatment of MDD are largely speculative. While many authors are unable to detect any sex-based differences of the efficacy of ADDs, others state superiority when used in either males or females. However, findings are inconsistent (Sramek et al. 2016). It appears that SSRIs and to a lesser extent SSNRIs may be superior in the treatment of women. Men, on the other hand, may respond better to TCAs (Sramek et al. 2016). In a meta-analysis including 15 studies, Khan et al. examined sex-specific treatment response. While both sexes showed significant improvement, women improved to a greater extent than men when treated with an SSRI, while treatment with an SSNRI showed similar responses among sexes (Khan et al. 2005). Unfortunately, most of these studies do not take the occurrence of ADRs into account (Sramek et al. 2016) which are often pivotal in the acceptance of any medication (Sansone and Sansone 2012).

Serotonergic ADRs often caused by SSRIs and SSNRIs may induce or worsen pre-existing sexual dysfunction (Montejo et al. 2019b). While sexual dysfunction as an ADR affects men and women equally, it may be more poorly tolerated by men (Montejo et al. 2019a) resulting in cessation of the drug or decreased willingness to start treatment in the first place. Bupropion and mirtazapine are associated with a lower incidence of sexual dysfunction (Montejo et al. 2019b) and may therefore be preferred by men. This may in part explain the higher utilization of mirtazapine, and even more significantly bupropion, as found in this study.

Higher utilization of lp FGAs by women and SGAs by men was consistent both in 2001–2017 and 2015–2017. A reason for this may also be the underlying clinical considerations regarding potential ADRs. Weight gain is frequently associated with certain psychotropic drugs, especially SGAs, such as olanzapine and quetiapine, but also ADDs, such as mirtazapine (Schneider et al. 2020), which each showed higher utilization among men at some point during this study’s observation period. Mirtazapine and olanzapine showed sex differences during the overall observation period. Initially in 2001–2003, mirtazapine had similar utilization rates among sexes with significant differences in utilization in 2015–2017. At this time, quetiapine also showed higher use among men, which was not the case in the overall observation period or in 2001–2003. Among drugs with the greatest risk for weight gain, quetiapine showed the smallest sex difference overall and also in 2015–2017, perhaps because quetiapine (extended release) is the only APD that is in-label for the treatment of MDD according to German guidelines (DGPPN et al., 2017).

Parallel to the increased use of weight gain-inducing psychotropic drugs by men, utilization of HYPDs and TRDs showed more discrepant sex differences in 2015–2017 alongside agomelatine and trazodone—all of which are ascribed a lower risk of weight gain while also having sedating and/or sleep-promoting properties (Fachinfo-Service 2020). Perhaps this indicates that women were even less willing to tolerate potential weight gain during the later timeframe. This may not be without reason as women may be more susceptible to psychotropic drug-induced weight gain (Seeman 2009, 2020; Tandon et al. 2020) and have a harder time subsequently losing gained weight (Seeman 2009).

### Sex differences in biology and pharmacokinetics of psychotropic drugs

Research suggests that sex strongly interacts with many brain function due to hormonal effects and effects of genomic sex (i.e., presence of two X-chromosomes or one X- and one Y-chromosome) (Rubinow and Schmidt 2019). These processes, though not understood in full, provide possible explanations for the higher incidence of MDD in females (Rubinow and Schmidt 2019) and are also postulated to have relevant effects on the treatment of depression (Hernández-Hernández et al. 2019). Progesterone, for example, has shown to induce an enhanced receptor binding of benzodiazepines making these drugs more potent when used in the treatment of women (Farkouh et al. 2020). This may present a possible explanation for the higher use of benzodiazepines in the treatment of women in this patient collective.

Furthermore, there are sex-related differences in the metabolism of drugs via cytochrome P450 (CYP) enzymes. CYP3A4 is well-researched on this behalf and has been found to be up to 50% more active in adult Caucasian women (Farkouh et al. 2020), while CYP1A2 activity is higher in males (Scandlyn et al. 2008). It seems rather unlikely that the consideration of CYP-enzyme profiles according to the patient’s sex played a relevant role in a clinician’s choice to prescribe a certain drug or refrain from doing so. However, activity of CYP-enzymes plays a decisive role in how a drug is metabolized and lastly how well it is tolerated by a patient. These implications may have led clinicians to observe that certain drugs are tolerated better when used in the treatment of either men or women. Under consideration of their pharmacokinetic profiles, these observations can be applied to some of the respective isoenzymes’ substrates in this study. Trazodone, a substrate of CYP3A4 (Procyshyn et al. 2019), was used more in the treatment of female patients in this study, while the CYP1A2 substrate olanzapine (Procyshyn et al. 2019) showed higher use among males. This does not hold true for all sex differences observed among psychotropic drugs. For example, duloxetine is primarily metabolized via CYP1A2, while the major pharmacokinetic pathways of mirtazapine include CYP3A4 (Procyshyn et al. 2019). Consideration of activity of CYP-enzymes according to the above-mentioned sex differences alone will not guarantee a successful drug treatment—for this purpose, CYP testing to detect inter-individual variability in drug response remains the gold standard (Samer et al. 2013).

### Sex differences in help-seeking behavior, symptom presentation, and polypsychopharmacy

Epidemiological studies on the prevalence of MDD consistently report that women have a two-fold increased risk of being diagnosed with MDD (Herzog et al. 2019). The higher willingness of women to seek professional help for their emotional problems is well-known (Frackiewicz et al. 2000). This of course does not apply in the same extent to the inpatients in this collective who are already in psychiatric treatment, but it may be one possible explanation for the higher use of psychotropic drugs among women in population-based settings. If men are more hesitant to report symptoms of MDD to a physician, concomitant psychotropic drug use arising from symptoms such as sleeping disorders would also be expected to be lower in the inpatient setting.

In general, women with MDD are three times more likely to present with atypical symptoms (e.g., anxiety, irritability, increased appetite) (Halbreich and Kahn 2007). Females are more likely to ruminate, complain of somatic discomfort, such as autonomic cardio-respiratory and gastrointestinal symptoms, and suffer from comorbid psychiatric diagnoses, such as anxiety, panic, and eating disorders (Gorman 2006; Halbreich and Kahn 2007). Somatic symptoms have shown to preferentially respond to SSRIs (LeGates et al. 2019), however, these manifestations may not be sufficiently alleviated by treatment with an ADD alone, therefore leading to the prescription of other psychotropic drugs. This may explain why female patients in this study, but also in other studies (Boyd et al. 2015), are more likely to be prescribed a higher number of psychotropic drugs, such as TRDs and HYPDs, which were both more frequently used in the treatment of women, as well as pregabalin — an AED primarily used for its anxiolytic and not its mood-stabilizing properties. Men, on the other hand, are more likely to externalize symptoms and report substance misuse, impulsivity, over-involvement in work (Oliffe et al. 2019), and tension within interpersonal relationships (Altemus et al. 2014)—symptoms which are not included by diagnostic criteria resulting in frequent oversight (Oliffe et al. 2019).

While female SSRI and NaSSA users were both more likely to simultaneously use HYPDs and TRDs, this study was unable to identify comparable sex differences among SSNRI users. This may indicate that SSNRIs cause more psychiatric ADRs, such as agitation, sleep disorders, and anxiety, in general which in turn raise the demand for symptom-driven prescription of sedating drugs, such as HYPDs and TRDs, regardless of sex. This study found that male venlafaxine users were more likely to concomitantly utilize other psychotropic drugs with sedating properties, such as mirtazapine and SGAs, than their female counterparts, perhaps again indicating that men are more willing to utilize weight gain-inducing drugs to achieve sedating effects.

Apart from the use of psychotropic drugs, the treatment of MDD includes numerous non-pharmacological treatment strategies, such as psychotherapy and electroconvulsive therapy (ECT). A minority (i.e., 1.72%) of patients with affective disorders in Germany are prescribed ECT (Timäus et al. 2021). Females have been reported to more often receive ECT (Timäus et al. 2021; Buley et al. 2017; Wood and Burgess 2003), while at the same time, recipients of ECT are treated with a substantially higher number of ADDs, APDs, HYPDs, and TRDs (Wilkinson et al. 2018). Psychotherapy, on the other hand, is much more commonly encountered treatment method. Outpatient data from Germany suggest that women are more likely to receive psychotherapy (Epping et al. 2017). The combined use of psychotherapy and pharmacotherapy has been associated with superior outcomes in the treatment of MDD than either strategy alone (Cuijpers et al. 2014). While data on concurrent ECT and psychotherapy are not available for this patient collective, these findings from other studies may further implicate an increased willingness of females to seek and acquire mental health care both of pharmacological and non-pharmacological entity.

## Limitations

The data presented here only represent a descriptive analysis of the current state of drug utilization based on sex during a 17-year time period. Other clinically relevant aspects also potentially significantly determining the selection of psychotropic drugs, such as patient age, (psychiatric) comorbidity, and severity of MDD, were not considered in this study. As collected data and reported diagnoses are based on routine clinical data, certain aspects (e.g., somatic diagnoses, other sociodemographic characteristics) were not fully documented. Information on the use of non-pharmacological treatment options, such as psychotherapy and ECT, is not accessible. Further, data regarding clinical aspects of treatment, such as course of clinical symptoms and the occurrence of ADRs, as well as (psychiatric) comorbidities indicating the use of a psychotropic drug (such as AEDs for epilepsy) was not available. Therefore, the rationale behind the selection of a certain drug/drug group was not verifiable for this collective of inpatients. It would also not be retraceable, if the utilization of multiple drugs was due to cross-tapering strategies to some extent when switching from one psychotropic drug to another, thus resulting in an overestimation of utilization rates and polypsychopharmacy.

## Conclusion

This study examining the psychopharmacological treatment of inpatients with MDD detected several relevant differences between men and women from 2001 to 2017. Monotherapy was more common among male patients, whereas women had a higher risk of being treated with four or more psychotropic drugs. Men and women were equally likely to be treated with ADDs. Utilization of NaSSAs (mainly mirtazapine) and bupropion was higher among men, whereas other ADD-subgroups (e.g., SSRIs and TCAs) showed only minimal sex differences. APDs were used in the treatment of almost half of both men and women, with an increasing utilization trend in 2015–2017, especially among men. Men had higher use of SGAs, whereas women were more likely to use lp FGAs. Use of TRDs and HYPDs showed a consistently higher utilization in female inpatients, which was even more pronounced for HYPD use among women in 2015–2017.

The clinical data presented here show that men and women with MDD are treated differently in terms of psychopharmacological approaches. While the sex differences found in this study are not as large as found by other authors among the ambulatory setting, they are clearly detectable, especially in the later timeframe of this study (i.e., 2015–2017). This indicates that the wide-spread discussion of the effect of sex is also finding consideration in the treatment of MDD in naturalistic, clinical settings. Current research suggests that sex differences in the development and treatment of MDD may be of unprecedented importance. Sex-specific recommendations in the treatment of MDD are yet to be made. Further research is needed to validate and better comprehend sex differences in the treatment response to psychotropic drugs.
